# ADAMTS13 activity as a novel risk factor for incident type 2 diabetes mellitus: a population-based cohort study

**DOI:** 10.1007/s00125-016-4139-5

**Published:** 2016-10-27

**Authors:** Paul S. de Vries, Thijs T. W. van Herpt, Symen Ligthart, Albert Hofman, M. Arfan Ikram, Mandy van Hoek, Eric J. G. Sijbrands, Oscar H. Franco, Moniek P. M. de Maat, Frank W. G. Leebeek, Abbas Dehghan

**Affiliations:** 1 Department of Epidemiology, Erasmus University Medical Center, Rotterdam, the Netherlands; 2 Human Genetics Center, University of Texas Health Science Center at Houston, Houston, TX USA; 3 Department of Internal Medicine, Erasmus University Medical Center, Rotterdam, the Netherlands; 4 Department of Neurology, Erasmus University Medical Center, Rotterdam, the Netherlands; 5 Department of Radiology, Erasmus University Medical Center, Rotterdam, the Netherlands; 6 Department of Hematology, Erasmus University Medical Center, Rotterdam, the Netherlands; 7 Department of Epidemiology and Biostatistics, Imperial College London, St Mary’s Campus, Norfolk Place, London, W2 1PG UK

**Keywords:** ADAMTS13, Diabetes, Epidemiology, Incidence, Prediabetes, Risk factor, Von Willebrand factor

## Abstract

**Aims/hypothesis:**

ADAMTS13 is a protease that breaks down von Willebrand factor (VWF) multimers into smaller, less active particles. VWF has been associated with an increased risk of incident type 2 diabetes mellitus. Here, we determine whether ADAMTS13 activity and VWF antigen are associated with incident diabetes.

**Methods:**

This study included 5176 participants from the Rotterdam Study, a prospective population-based cohort study. Participants were free of diabetes at baseline and followed up for more than 20 years. Cox proportional hazards models were used to examine the association of ADAMTS13 activity and VWF antigen with incident diabetes.

**Results:**

ADAMTS13 activity was associated with an increased risk of incident diabetes (HR 1.17 [95% CI 1.08, 1.27]) after adjustment for known risk factors and VWF antigen levels. Although ADAMTS13 activity was positively associated with fasting glucose and insulin, the association with incident diabetes did not change when we adjusted for these covariates. ADAMTS13 activity was also associated with incident prediabetes (defined on the basis of both fasting and non-fasting blood glucose) after adjustment for known risk factors (HR 1.11 [95% CI 1.03, 1.19]), while the VWF antigen level was not. VWF antigen was associated with incident diabetes, but this association was attenuated after adjustment for known risk factors.

**Conclusions/interpretation:**

ADAMTS13 activity appears to be an independent risk factor for incident prediabetes and type 2 diabetes. As the association between ADAMTS13 and diabetes did not appear to be explained by its cleavage of VWF, ADAMTS13 may have an independent role in the development of diabetes.

**Electronic supplementary material:**

The online version of this article (doi:10.1007/s00125-016-4139-5) contains peer-reviewed but unedited supplementary material, which is available to authorised users.

## Introduction

A disintegrin and metalloprotease with a thrombospondin type 1 motif, member 13 (ADAMTS13) reduces the activity of von Willebrand factor (VWF) in platelet adhesion and aggregation by cleaving prothrombotic VWF multimers [1, 2]. Low ADAMTS13 levels and activity are associated with an increased risk of various thrombotic diseases, including ischaemic stroke and myocardial infarction [3–8], as well as kidney disease [9]. Additionally, low ADAMTS13 activity may contribute to the renal and cardiovascular complications of diabetes [10–12]. However, the association of ADAMTS13 with diabetes itself remains unexplored. Elevated levels of VWF have been associated with an increased risk of type 2 diabetes [13–17] which has been attributed primarily to the role of VWF as a marker of endothelial dysfunction rather than to its role in thrombosis [18]. However, VWF may also be associated with diabetes through its prothrombotic effect. This would be in line with emerging evidence that vascular disease may contribute to the development of diabetes [19]. Low ADAMTS13 activity and high VWF levels may exacerbate small vessel disease, which in turn may contribute to the development of diabetes [20–22]. If VWF is associated with diabetes through its prothrombotic function, then we would expect ADAMTS13, with its antithrombotic function, to be inversely associated with the risk of diabetes. On the other hand, little is still known about the regulation of ADAMTS13 and its role as a marker of other physiological processes [23]. We previously showed that type 2 diabetes patients have higher ADAMTS13 activity compared with controls [8, 23], which is inconsistent with a mechanism involving VWF’s prothrombotic function.

Nevertheless, the association may also reflect a response to diabetes, and studies on incident diabetes are needed to provide further insight into the direction of this association. In this study, we examined whether ADAMTS13 activity or VWF antigen levels are associated with risk of type 2 diabetes in a large prospective population-based cohort study.

## Methods

### Study description and population

The Rotterdam Study is a prospective population-based cohort study initiated in 1990 to study the determinants of several chronic diseases in older adults [24]. The first cohort (RS-I) includes 7983 inhabitants of Ommoord, a district of Rotterdam in the Netherlands, who were aged ≥55 years at recruitment. The first examination took place between 1990 and 1993. The third visit, including 4797 participants, took place between March 1997 and December 1999, and was used as the baseline in this study. The second cohort (RS-II), established between February 2000 and December 2001, includes another 3011 inhabitants of Ommoord who either reached the age of 55 years after the recruitment phase of RS-I or had migrated into the research area. There were no eligibility criteria to enter the Rotterdam Study except for age and residential area (postal code). The Rotterdam Study was approved by the Medical Ethics Committee of the Erasmus Medical Center and the Ministry of Health, Welfare and Sport of the Netherlands, through implementing the Wet Bevolkingsonderzoek: ERGO (Population Studies Act: Rotterdam Study). All participants provided written informed consent to participate in the study and to obtain information from their treating physicians.

### Ascertainment of prediabetes and diabetes

**Fig. 1:**
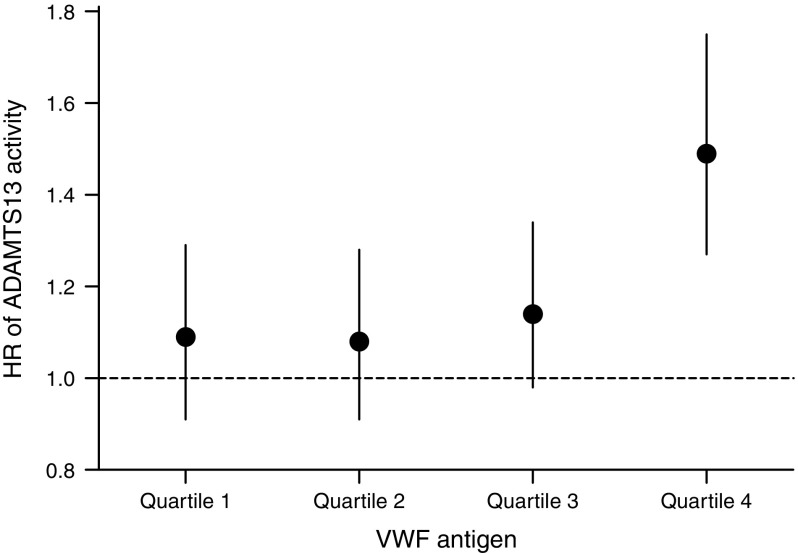
HRs of ADAMTS13 activity (per SD) for incident type 2 diabetes across quartiles of VWF antigen: interaction between ADAMTS13 and VWF. Vertical bars represent 95% CIs

Diabetes, prediabetes and normoglycaemia were defined according to the most recent WHO guidelines [25]. Prediabetes was defined as a fasting blood glucose level between 6.0 mmol/l and 7.0 mmol/l or a non-fasting blood glucose level between 7.7 mmol/l and 11.1 mmol/l (when fasting samples were not available); diabetes was defined as a fasting blood glucose level of >7.0 mmol/l, a non-fasting blood glucose level of ≥11.1 mmol/l (when fasting samples were not available), or the use of glucose-lowering medication. Prevalent and incident diabetes and prediabetes were ascertained using general practitioner records, hospital discharge letters, pharmacy records, home interviews and fasting glucose measurements performed at our research centre during baseline and follow-up visits [26]. Information regarding the use of blood glucose lowering medication was derived from both home interviews and pharmacy records [26]. A schematic representation of the follow-up design is provided in electronic supplementary material (ESM) Fig. 1. At baseline more than 99% of the Rotterdam Study population was covered by the pharmacies in the study area. All potential prediabetes and diabetes events were independently adjudicated by two study physicians; in the case of disagreement, consensus was sought with the help of an endocrinologist. Follow-up data is complete until 1 January 2012. Flowcharts detailing the reasons for classifying prevalent cases of diabetes and prediabetes are shown in ESM Figs 2 and 3, respectively.

### ADAMTS13 activity and VWF antigen measurements

Citrated plasma samples were collected at the third visit of RS-I and at the baseline examination of RS-II, and stored at −80°C. Between June and October 2013, we measured ADAMTS13 activity using a kinetic assay based on the fluorescence resonance energy transfer substrate VWF73 (FRETS-VWF73) assay [27]. Plasma samples were measured against a reference curve of serially diluted pooled normal human plasma defined as having an ADAMTS13 activity of 1 IU/ml, and we expressed ADAMTS13 activity as a percentage of this reference value. The ADAMTS13 activity of 6258 participants was measured: 3791 from RS-I and 2467 from RS-II.

Between July and October of 2008, VWF antigen levels (in IU/ml) were determined with an in-house ELISA using polyclonal rabbit anti-human VWF antibodies (DakoCytomation, Glostrop, Denmark) for capturing and tagging [28]. The intra-assay CV was 5.8% and the interassay CV was 7.8%. VWF antigen levels were measured in 3968 individuals from RS-I and in 2561 individuals from RS-II.

In total, 5176 participants with VWF and ADAMTS13 measurements also had a fasting glucose measurement and were free of diabetes at baseline, while 4232 participants were free of prediabetes at baseline (ESM Fig. 2 and 3).

### Covariates

BMI was calculated by dividing the weight in kg by the height in metres squared. Information on current tobacco smoking was acquired from questionnaires. Lipid-lowering (statins, fibrates, and other lipid modifying agents), antihypertensive (diuretics, beta blockers, ACE inhibitors, calcium channel blockers) and antithrombotic medication use was assessed during a structured home interview. Blood pressure was measured twice using an oscillometric device after 5 min of rest, and the mean value was taken as the final reading. Serum total cholesterol and HDL-cholesterol levels were determined using an automated enzymatic method. Blood glucose levels were measured using the glucose hexokinase method (Instruchemie, Delfzijl, the Netherlands) [29]. Insulin levels were determined by metric assay (BioSource Diagnostics, Camarillo, CA, USA). This assay does not cross-react with either proinsulin or C-peptide. Serum alanine aminotransferase (ALT) levels were measured using a Merck Diagnostica kit (Whitehouse Station, NJ, USA) on an Elan Autoanalyzer (Merck). White blood cell counts were assessed in citrate plasma with a Coulter Counter T540 (Coulter Electronics, Hialeah, Florida, USA). C-reactive protein (CRP) was measured using CRPL3, an immunoturbidimetric assay (Roche Diagnostics, Indianapolis, IN, USA). Prevalent CHD was defined as having a history of myocardial infarction or coronary revascularisation procedures, as previously described [30].

### Statistical analysis

Statistical analyses were performed using IBM SPSS Statistics software (version 21.0, Armonk, NY, USA) and R (version 3.1.3; R Foundation for Statistical Computing, Vienna, Austria). Missing values for covariates (<5%) were imputed in SPSS using single imputation based on expectation maximisation. VWF antigen, HDL-cholesterol, CRP, ALT and fasting insulin levels were natural log transformed. We used linear regression models to test the association of ADAMTS13 activity and VWF antigen level with fasting glucose and fasting insulin levels. Individuals with prevalent diabetes were excluded from all analyses.

The association of ADAMTS13 activity and VWF antigen with incident diabetes was examined using Cox proportional hazards models. Three adjustment models were used: Model 1 was adjusted for age, sex and cohort; Model 2 was additionally adjusted for HDL and total cholesterol, lipid-lowering medication, BMI, CRP, current smoking, antithrombotic medication, ALT, white blood cell count, systolic BP, antihypertensive medication and prevalent CHD; and Model 3 was additionally adjusted for fasting glucose and insulin. The assumption of proportional hazards was met for all models. In Model 1 ADAMTS13 activity and VWF antigen were tested separately, whereas in Models 2 and 3 the analysis of ADAMTS13 activity was adjusted for VWF antigen level and vice versa. We examined the interaction between ADAMTS13 activity and VWF antigen on incident diabetes using a multiplicative interaction term, and adjusting for age, sex and cohort. Results are shown per SD of VWF antigen levels and ADAMTS13 activity. We also tested the association of ADAMTS13 activity quartiles with incident diabetes.

To test whether associations with incident diabetes were driven by participants with prevalent CHD, or users of lipid-lowering, antihypertensive and antithrombotic medication, we excluded participants in each of these subgroups in a sensitivity analysis.

## Results

Baseline characteristics are shown in Table 1. In a median follow-up of 11.2 years (interquartile range 9.8–12.6), 638 of the 5176 participants who were free of diabetes at baseline developed diabetes (incidence rate 12.4 per 1000 person-years). As shown in ESM Fig. 4, 36 of these individuals were diagnosed during follow-up because they started with insulin treatment, whereas 278 started using oral glucose-lowering medication. Another 324 participants were diagnosed with diabetes because of their high fasting glucose levels. Of the 4232 participants without prevalent prediabetes, 862 developed prediabetes (incidence rate 21.1 per 1000 person-years). During follow-up, 13 of these individuals started insulin treatment, 118 started taking oral glucose-lowering medication and 731 were diagnosed with prediabetes based on their fasting glucose levels (shown in ESM Fig. 5).

**Table 1:** Baseline characteristics of the study population

Characteristic	Value
Age (years)	69.0 ± 8.1
Sex (female)	57.7
BMI (kg/m^2^)	26.7 ± 3.8
HDL-cholesterol (mmol/l)	1.3 (1.12–1.60)
Total cholesterol (mmol/l)	5.9 ± 1.0
Lipid-lowering medication use	11.4
Systolic BP (mmHg)	142.1 ± 21.0
Antihypertensive medication use	20.8
ALT (U/l)	20.0 (16.0–26.0)
Former smoker	40.7
Current smoker	12.5
CRP (nmol/l)	21.0 (10.0–44.3)
White blood cell count (×10^9^ cells/l)	6.7 ± 1.9
Prevalent CHD	7.3
Prevalent prediabetes	18.2
Fasting glucose level (mmol/l)	5.5 ± 0.5
Fasting insulin level (pmol/l)	71.36 (50.60–100.00)
Antithrombotic medication use	17.4
ADAMTS13 activity (%)	91.0 ± 17.2
VWF antigen level (IU/ml)	1.3 ± 0.6

*N* = 5176

Data are the mean ± SD, percentage or median (interquartile range)

Associations of ADAMTS13 activity and VWF antigen level with incident diabetes are shown in Table 2. ADAMTS13 activity was associated with a 19% increased risk of incident diabetes per SD in the age- and sex-adjusted model (HR 1.19 [95% CI 1.10, 1.30]), and this association remained unchanged after adjusting for potential confounders. Participants in the highest quartile of ADAMTS13 activity had a 46% increased risk compared with participants in the lowest quartile (HR 1.46 [95% CI 1.15, 1.85]). Increased VWF antigen level was associated with a 12% (HR 1.12 [95% CI 1.03, 1.21]) increased risk of incident diabetes per SD in the age- and sex-adjusted model. However, the increased risk was attenuated to 6% (HR 1.06 [95% CI 0.98, 1.15]) increased risk per SD after adjustment for additional covariates, and was 8% (HR 1.08 [95% CI 0.99, 1.17]) after further adjustment for fasting glucose and insulin levels.

**Table 2:** HRs for ADAMTS13 activity on incident type 2 diabetes

ADAMTS13 activity	Model 1		Model 2		Model 3	
	HR (95% CI)	*p* value	HR (95% CI)	*p* value	HR (95% CI)	*p* value
Continuous (per SD)	1.19 (1.10, 1.30)	0.00003	1.17 (1.08, 1.27)	0.0001	1.17 (1.08, 1.27)	0.00009
Quartile 1 (*N*_cases_: 129)	Reference		Reference		Reference	
Quartile 2 (*N*_cases_: 150)	1.12 (0.88, 1.42)	0.4	1.10 (0.87, 1.39)	0.4	1.12 (0.88, 1.41)	0.4
Quartile 3 (*N*_cases_: 168)	1.26 (1.00, 1.59)	0.05	1.31 (1.03, 1.65)	0.03	1.36 (1.08, 1.73)	0.01
Quartile 4 (*N*_cases_: 191)	1.47 (1.16, 1.86)	0.001	1.46 (1.15, 1.85)	0.002	1.48 (1.17, 1.88)	0.001

Model 1 was adjusted for age, sex and cohort; Model 2 was additionally adjusted for VWF antigen, HDL and total cholesterol, lipid-lowering medication, body-mass index, CRP, former smoking, current smoking, antithrombotic medication, ALT, white blood cell count, systolic BP, antihypertensive medication and prevalent CHD; and Model 3 was additionally adjusted for glucose and insulin levels

HDL-cholesterol, CRP, ALT and insulin were natural log transformed before use

*N*_cases_, number of incident diabetes cases

Both ADAMTS13 activity and VWF antigen level were positively associated with the baseline fasting insulin level, and ADAMTS13 activity was positively associated with the baseline fasting glucose level (ESM Table 1). Nevertheless, when we additionally adjusted for fasting glucose and insulin levels, the effect sizes did not change. These associations were robust to the exclusion of participants with prevalent CHD at baseline and to the exclusion of users of lipid-lowering, antihypertensive and antithrombotic medications (ESM Table 2). There was an interaction between ADAMTS13 activity and VWF antigen level with incident diabetes (*p* = 0.01). As shown in Fig. 1, the association of ADAMTS13 activity with incident diabetes was strongest in the fourth quartile of VWF antigen level (HR 1.49 [95% CI 1.27, 1.75]).

**Fig. 2:**
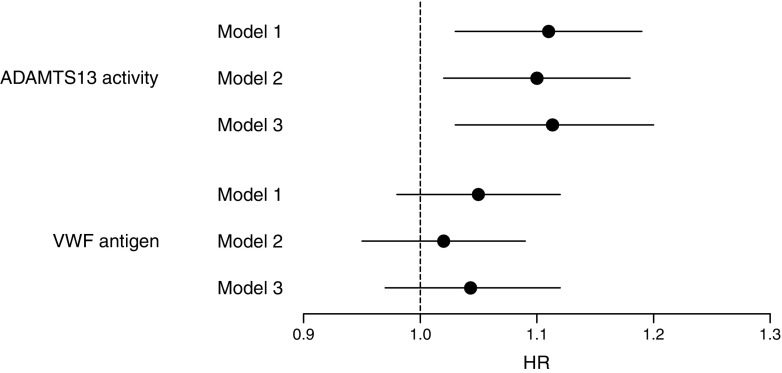
HRs of ADAMTS13 activity and natural log transformed VWF antigen (per SD) for incident prediabetes, excluding participants with prediabetes at baseline (862 events in 4232 participants). Horizontal bars represent 95% CIs

Furthermore, ADAMTS13 activity was also associated with an 11% (HR 1.11 [95% CI 1.03, 1.19]) increased risk of prediabetes per SD in Model 1, and this association was similar in Models 2 and 3 (Fig. 2). In contrast, VWF antigen level was not associated with incident prediabetes.

## Discussion

In our study, ADAMTS13 activity was associated with an increased risk of incident diabetes, even after adjustment for other known risk factors, including VWF antigen, fasting glucose and fasting insulin levels. Furthermore, ADAMTS13 activity was associated with the incidence of prediabetes among participants with normoglycaemia at baseline. The VWF antigen level was also associated with an increased risk of diabetes, but this association was attenuated after adjustment for known risk factors. These results suggest a role for ADAMTS13 in the occurrence of type 2 diabetes at an early stage before glucose levels rise.

To our knowledge, the association of ADAMTS13 with diabetes has not previously been studied with diabetes used as the primary outcome, and we are the first to examine this association in a large prospective population-based cohort study. One cross-sectional study reported an association between ADAMTS13 and prevalent diabetes [12]. These researchers did not observe a significant difference in ADAMTS13 levels between 86 diabetes patients and 26 healthy controls. We previously observed that ADAMTS13 activity is 5% higher in participants with prevalent diabetes compared with those without prevalent diabetes [31]. In the current study, we excluded participants with prevalent diabetes, and instead focused on the risk of future diabetes. The results of this study are therefore largely independent of the results of the previous cross-sectional study.

Our results for VWF are consistent with those of previous studies. VWF has been associated with incident diabetes in a range of studies [13–17], but in general the association weakened after adjustment for confounders and became non-significant. In the Framingham Heart Study, however, VWF remained significantly associated with incident diabetes after adjustment for a wide range of potential confounders, including insulin resistance [14]. Similarly, in the Coronary Artery Risk Development in Young Adults study, VWF remained associated with insulin resistance after multivariable adjustment [32]. VWF is a marker of endothelial dysfunction, which is thought to explain the association between VWF levels and diabetes [18]. We report an interaction between ADAMTS13 activity and VWF, with the largest effect of ADAMTS13 activity in participants within the highest quartile of VWF levels. This interaction suggests that the effect of ADAMTS13 is mainly present in individuals with advanced endothelial dysfunction.

The mechanism underlying the association of ADAMTS13 activity with diabetes remains unclear. The findings that the association was robust to the adjustment for baseline fasting glucose and insulin levels and that ADAMTS13 activity was also associated with incident prediabetes limit the possibility of reverse causation. The latter association, for example, suggests that high ADAMTS13 activity in individuals with healthy glucose metabolism is associated with development of the early subclinical stages of type 2 diabetes. However, the association between ADAMTS13 activity and diabetes is unlikely to be explained by its only robustly identified function as a cleaving protease of VWF, because in that case we would expect VWF level (prothrombotic) and ADAMTS13 activity (antithrombotic) to be associated with diabetes in opposite directions. An alternative hypothesis is that ADAMTS13 has an additional proteolytic functionality beyond VWF cleavage. After an initial interaction with globular VWF, ADAMTS13 undergoes a conformational change that increases its ability to break down VWF [33]. Recent research suggests that this conformational change increases the ability of ADAMTS13 to break down not only VWF but also other proteins such as fibrinogen [34]. ADAMTS13 activity, as measured in our study, may partly reflect this process. As such, the observed association between ADAMTS13 activity and incident type 2 diabetes might be explained by the interaction of ADAMTS13 with one or more currently unknown proteins. Finally, the association could be explained by pathways responding to ADAMTS13. For example, there is preliminary evidence that ADAMTS13 upregulates vascular endothelial growth factor, a protein involved in angiogenesis that may contribute to the development of type 2 diabetes [35, 36]. ADAMTS13 may similarly activate other pathways that lead to the development of type 2 diabetes. However, since its discovery in 2001, most ADAMTS13 research has focused on its interactions with VWF and its role in thrombotic thrombocytopenic purpura. Therefore, we believe that further research is required to elucidate other pathways affected by ADAMTS13.

We measured ADAMTS13 activity using the FRETS-VWF73 assay, which is based on a synthetic peptide spanning the VWF cleavage site [27]. An alternative is to measure ADAMTS13 antigen levels, which correspond to the abundance of ADAMTS13. Future studies should investigate whether ADAMTS13 activity or antigen level is most strongly associated with diabetes. If the association with diabetes is strongest with ADAMTS13 antigen, then the association of markers of *ADAMTS13* gene expression and of ADAMTS13 synthesis, secretion and degradation with diabetes should be explored. Alternatively, a stronger association with ADAMTS13 activity implicates a factor downstream of VWF cleavage and not decreased VWF activity itself.

The strengths of our study include the comprehensive assessment of incident diabetes and prediabetes using medical records, links with pharmacies in the study area and standardised blood glucose measurement at each follow-up visit. Additionally, we used data from a well-characterised prospective population-based cohort study, which enabled us to correct for a wide range of covariates. We used a long follow-up and adjusted for baseline fasting glucose and insulin levels to reduce the possibility of reverse causation. By also examining associations with incident prediabetes, we provide insight into the early development of subclinical disease.

The main limitation of our study is that, as for all observational studies, we cannot rule out residual confounding. In addition, we included individuals aged ≥55 years and effect estimates might not be generalisable to younger individuals. Another limitation of this study is that we did not measure VWF activity or the ratio of small inactive VWF to large active VWF. Such measurements would provide insight into whether the observed association is related to the proteolysis of VWF by ADAMTS13.

In conclusion, we identified ADAMTS13 activity as a novel independent marker of incident diabetes that is associated with both diabetes and prediabetes. Further research is necessary to confirm this association and to elucidate the biological mechanism underlying this association. Exploration of alternative mechanisms of ADAMTS13 beyond VWF cleavage is warranted because the association may not be explained by its antithrombotic function.

## Electronic supplementary material

Below is the link to the electronic supplementary material.

ESM 1 (PDF 157 kb)

## Abbreviations

ADAMTS13: A disintegrin and metalloprotease with a thrombospondin type 1 motif member 13
ALT: Alanine aminotransferase
CRP: C-reactive protein
FRETS-VWF73: Fluorescence resonance energy transfer substrate VWF73
RS-I: Rotterdam Study cohort 1
RS-II: Rotterdam Study cohort 2
VWF: Von Willebrand factor
