# Supplementary material for: ADAMTS13 activity as a novel risk factor for incident type 2 diabetes mellitus: a population-based cohort study
Source: Diabetologia. 2016 Oct 27;60(2):280–6. doi: 10.1007/s00125-016-4139-5 (PMC6518068; doi:10.1007/s00125-016-4139-5)

Electronic Supplementary Material (ESM)

ESM Table 1: Cross-sectional association of ADAMTS13 activity and VWF antigen (per SD) with fasting glucose and natural log transformed fasting insulin.

	ADAMTS13 activity		VWF antigen	
	β coefficient (95%CI)	*p* value	β coefficient (95%CI)	*p* value
Glucose				
Model 1	0.03 (0.01, 0.04)	0.001	0.01 (-0.00, 0.03)	0.08
Model 2	0.02 (0.01, 0.04)	0.003	-0.01 (-0.02, 0.01)	0.4
Insulin				
Model 1	0.06 (0.04, 0.07)	6×10^{-15}	0.07 (0.05, 0.08)	1×10^{-19}
Model 2	0.05 (0.04, 0.06)	4×10^{-15}	0.03 (0.02, 0.05)	2×10^{-8}

β coefficient refers to the 1 unit increase in fasting glucose (mmol/l) or insulin (natural log transformed pmol/l) per 1 standard deviation increase in VWF antigen or ADAMTS13 activity. *Adjustments:* Model 1: Adjusted for age, sex, and cohort. Model 2: Additionally adjusted for HDL and total cholesterol, lipid-lowering medication, body-mass index, CRP, former smoking, current smoking, antithrombotic medication, ALT, white blood cell count, systolic blood pressure, antihypertensive medication, and prevalent CHD. The analysis of VWF antigen was adjusted for ADAMTS13 activity and vice versa. HDL cholesterol, CRP, and ALAT were natural log transformed.

ESM Table 2: Association of ADAMTS13 activity and VWF antigen (per SD) with incident diabetes after exclusions based on disease and medication use at baseline*.

	ADAMTS13 Activity		VWF antigen	
	HR (95%CI)	*p* value	HR (95%CI)	*p* value
Excluding cases of prevalent CHD: 565 events in 4674 participants				
Model 1	1.20 (1.10, 1.30)	0.00007	1.14 (1.05, 1.25)	0.002
Model 2	1.17 (1.08, 1.28)	0.0003	1.09 (1.00, 1.19)	0.06
Model 3	1.18 (1.09, 1.29)	0.0001	1.10 (1.00, 1.20)	0.04
Excluding antithrombotic medication users: 490 events in 4062 participants				
Model 1	1.20 (1.09, 1.32)	0.0001	1.14 (1.04, 1.25)	0.006
Model 2	1.16 (1.06, 1.27)	0.002	1.08 (0.98, 1.18)	0.1
Model 3	1.16 (1.06, 1.27)	0.001	1.09 (0.99, 1.20)	0.08
Excluding lipid-lowering medication users: 526 events in 4372 participants				
Model 1	1.18 (1.08, 1.29)	0.0005	1.12 (1.03, 1.23)	0.01
Model 2	1.15 (1.05, 1.26)	0.002	1.06 (0.96, 1.15)	0.2
Model 3	1.15 (1.05, 1.26)	0.002	1.07 (0.97, 1.17)	0.2
Excluding antihypertensive medication users: 415 events in 3837 participants				
Model 1	1.19 (1.07, 1.32)	0.001	1.12 (1.01, 1.24)	0.03
Model 2	1.20 (1.08, 1.33)	0.0005	1.07 (0.96, 1.18)	0.2
Model 3	1.20 (1.08, 1.33)	0.0005	1.12 (1.01, 1.24)	0.04

*Exclusions were based on non-imputed variables.

Adjustments: Model 1: Adjusted for age, sex, and cohort. Model 2: Additionally adjusted for HDL and total cholesterol, lipid-lowering medication, body-mass index, CRP, former smoking, current smoking, antithrombotic medication, ALT, white blood cell count, systolic blood pressure, antihypertensive medication, and prevalent CHD. The analysis of VWF antigen was adjusted for ADAMTS13 activity and vice versa. Model 3: Additionally adjusted for glucose and insulin levels. HDL cholesterol, CRP, ALAT, and insulin were natural log transformed when used.

ESM Figure 1: Schematic representation of the follow-up design.

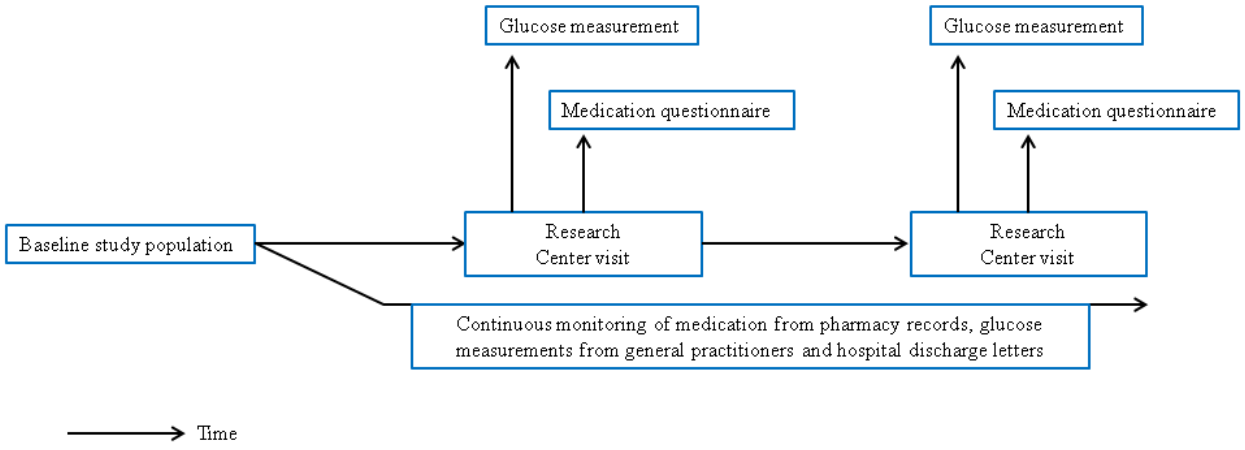

ESM Figure 2: Selection of the study population at baseline for incident diabetes follow-up.

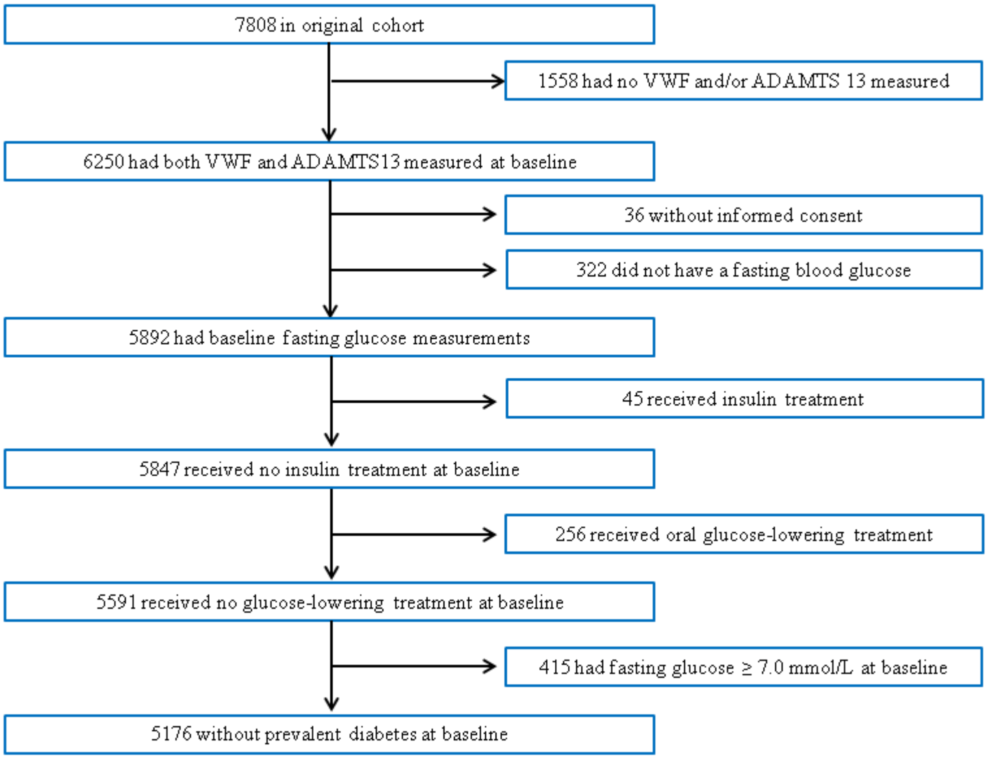

ESM Figure 3: Selection of the study population at baseline for incident prediabetes follow-up.

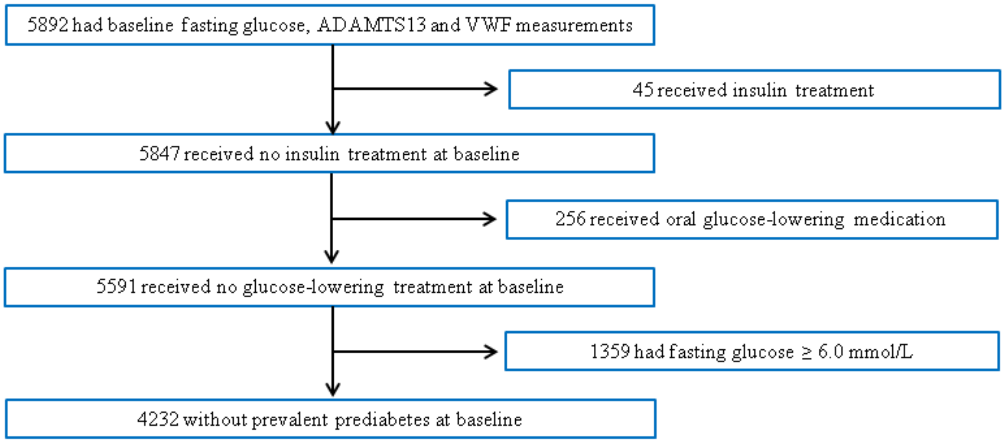

ESM Figure 4: Follow-up of incident diabetes events in the study population.

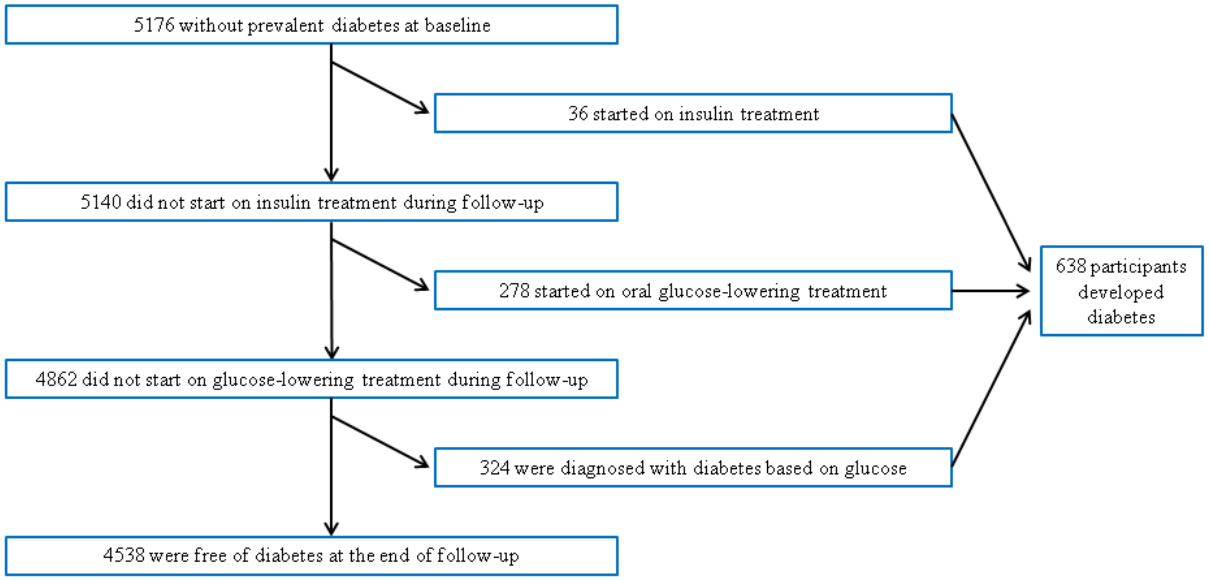

ESM Figure 5: Follow-up of incident prediabetes events in the study population.

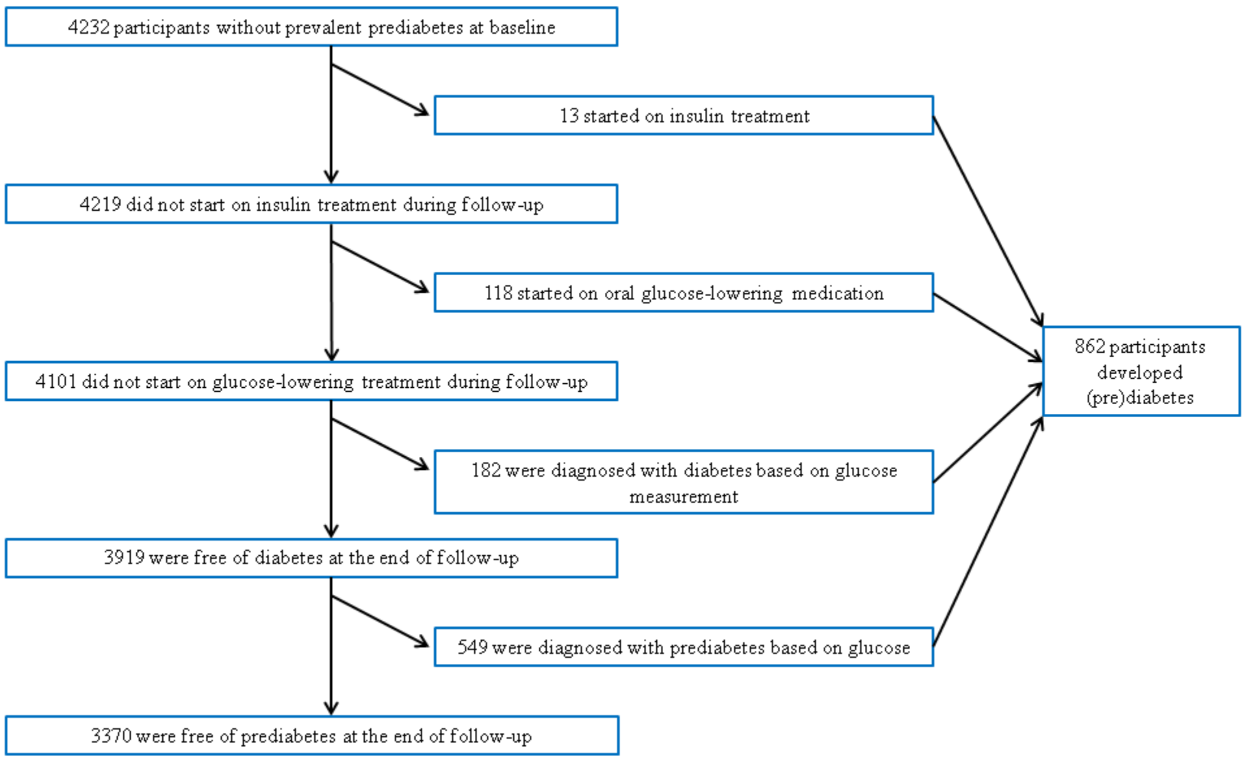

Supplement: Supplementary file 1 — (PDF 157 kb) [file 125_2016_4139_MOESM1_ESM.pdf]
